# European expert consensus on a structured approach to circular stapling anastomosis in minimally invasive left‐sided colorectal resection

**DOI:** 10.1111/codi.70037

**Published:** 2025-02-20

**Authors:** Samson Tou, Anthony G. Gallagher, Gabriele Bislenghi, Rui Farinha, Albert Wolthuis, the CAPITAL A Study Collaborative, Felix Aigner, Nicola Eardley, Christina Fleming, Dieter Hahnloser, Seon‐Hahn Kim, Brendan Moran, Guglielmo Niccolò Piozzi, Gabrielle van Ramshorst, Rosa M. Jimenez Rodriguez, Ines Rubio‐Perez, Ioannis Virlos, Janindra Warusavitarne, Marek Zawadzki

**Affiliations:** ^1^ Department of Colorectal Surgery Royal Derby Hospital, University Hospitals of Derby and Burton NHS Foundation Trust Derby UK; ^2^ School of Medicine, Royal Derby Hospital, University of Nottingham Derby UK; ^3^ ORSI Academy Melle Belgium; ^4^ Department of Abdominal Surgery University Hospital Leuven Leuven Belgium

**Keywords:** anastomosis, circular stapling, colorectal resection, colorectal surgery, metrics, minimally invasive surgery, proficiency‐based progression, training

## Abstract

**Aim:**

The aim of this work is to develop and operationally define performance metrics that characterize a reference approach to circular stapling anastomosis during minimally invasive left‐sided colorectal resection and to obtain face and content validity through a consensus meeting.

**Method:**

Three expert colorectal surgeons with advanced experience with minimally invasive surgery, a senior behavioural scientist and a research fellow with experience in performance metrics development formed the Metrics Group. Technical support was provided by device engineers. Published guidelines, training materials, manufacturers' instructions for use and unedited videos of circular stapling anastomosis in minimally invasive left‐sided colorectal resection were used to deconstruct the task into defined, observable performance units or metrics (i.e. procedural phases, steps, errors and critical errors). The performance metrics were then subjected to detailed review by 16 expert colorectal surgeons in a modified Delphi process.

**Results:**

Performance metrics for circular stapling anastomosis during minimally invasive left‐sided colorectal resection had three procedural phases with 32 steps, 40 errors and 38 critical errors. After the modified Delphi process the agreed performance metrics consisted of three procedural phases, 36 steps, 42 errors and 39 critical errors. A group of expert colorectal surgeons from Europe verified the face and content of these metrics. After discussion, all procedural phases received unanimous consensus by the Delphi panel.

**Conclusion:**

Circular stapling anastomosis during the minimally invasive approach to left‐sided colorectal resection can be broken down into procedural phases and steps, with errors and critical errors known as performance metrics. We consider the metrics essential for the development of structured training in using circular stapling anastomosis in the minimally invasive approach to left‐sided colorectal resection.

**What does this paper add to the literature?**

The present study is the first to describe the development and the performance metrics for training in circular stapling anastomosis for a minimally invasive approach to left‐sided colorectal resection.

## INTRODUCTION

The performance of intestinal anastomosis is one of the most critical steps in colorectal surgery, and complications associated with anastomosis can have devastating consequences for the patient's clinical, functional and oncological outcomes. Complications also create a significant burden on the healthcare system. Circular stapling is commonly performed in left‐sided colorectal anastomosis (sigmoid colectomy, high and low anterior resection) for benign and malignant conditions. It is used in open, laparoscopic and robotic surgeries. A recent review of a healthcare database with 13 167 patients who underwent left‐sided colorectal resection showed that 22.7% of patients had circular anastomotic complications [1]. In another study, knowledge gaps in many surgeons' understandings of the safe use of various commonly used medical devices, including stapling knowledge, were reported [2]. A high incidence of technical errors involving the use of circular staplers has also been reported [3]. Consequently, there is a need for surgical strategies and technologies to standardize and quality assure anastomotic techniques to lower the risk of anastomotic complications [4]. Emerging evidence has shown a strong relationship between the intraoperative performance of the surgeon operator and patient outcomes [5]. Our endeavour from a surgical community is to improve intraoperative performance [5], which we believe will have a considerable impact on patient safety and operative outcomes. One scientific approach to improving intraoperative performance is proficiency‐based progression (PBP) simulation training. PBP begins by deconstructing the procedure or skill being focused on into explicitly defined (binary) performance metrics, which are then validated [6]. The PBP approach to training makes skill acquisition more objective, transparent and fair. During training, trainees are given metric‐based feedback on their performance, which is explicit, constructive and formative [7]. In a recent systematic review of 12 prospective randomized and blinded clinical studies (PBP‐trained versus traditionally trained surgeons), PBP‐trained surgeons demonstrated significantly fewer performance errors (a 60% reduction) [8].

Our overarching goal was to improve training in circular stapling devices in minimally invasive left‐sided colorectal anastomosis using PBP methodology, and this first part of our project was to develop and objectively define performance metrics that characterize a reference approach to the application of circular stapling devices in left‐sided colorectal anastomosis during minimally invasive operations (i.e. laparoscopic and robotic) and to obtain face and content validity through a consensus meeting (i.e. with a Delphi panel) of very experienced and expert colorectal surgeons (senior consultant >10 years’ colorectal practice).

## METHOD

The principle of metric development and stress testing (face and content validation) for PBP training has been described in detail previously [9]. This approach was applied when developing the circular stapling anastomosis metrics for minimally invasive left‐sided colorectal anastomosis and is described below.

### Metrics Group

The Metrics Group consists of three experienced colorectal surgeons (AW, GB, ST) with a special interest in minimally invasive surgery, a senior behavioural scientist and an education–training expert (AGG), and a research fellow who is specialized in metrics development for surgical procedures (RF). Input was sought from device engineers who specialize in circular stapling devices.

### Circular stapling anastomosis metrics development

A detailed task analysis and deconstruction process was used to deconstruct a reference approach to the use of circular stapling anastomosis for minimally invasive left‐sided colorectal procedures in small, nonoverlapping performance units [10, 11, 12]. Published written guidelines, video teaching materials, manufacturer's instructions for use and access to 10 anonymized unedited minimally invasive left‐sided colorectal operations using circular stapling anastomosis performed by surgeons with different levels of experience supported the metrics development and procedure characterization process. The goal was to characterize a ‘reference’ approach to circular stapling anastomosis used in minimally invasive left‐sided colorectal operations. A reference procedure is assumed to be a straightforward and uncomplicated guide for trainees in learning the optimum performance of these procedures. The phases and steps are the same for female and male patients undergoing the anastomosis part of the minimally invasive left‐sided colorectal resection. For the ‘reference procedure’ there are agreed criteria for patient selection and procedure‐specific factors (Table 1).

**TABLE 1:** Patient selection criteria and procedure‐specific criteria for the ‘reference’ procedure.

Patient selection
Sigmoid colectomy
High/low anterior resection ± ileostomy
Body mass index 30 kg/m^2^ or less
American Society of Anesthesiologists grade 3 or less
Cancer or benign disease (e.g. diverticular disease)
Procedure
Minimally invasive (laparoscopic or robotic)
End‐to‐end anastomosis, double‐stapled anastomosis
Proximal end—well‐vascularized (indocyanine green optional), tension free (mobilized), transected perpendicular to its longitudinal axis
Distal end—well‐vascularized, transected perpendicular to its longitudinal axis

*Note*: A ‘reference’ procedure for training should be a straightforward, uncomplicated procedure.

A one‐day preliminary face‐to‐face planning meeting, three face‐to‐face meetings for metrics identification and definition and the metric stress test were conducted. Videoconferences (a total of 5 h) using Zoom (San Jose, CA, USA) and email exchanges were used to complement face‐to‐face meetings for further clarification and definition of the metrics.

At the beginning of the metrics development the Metrics Group agreed on the following definitions:

Performance metrics: units of observable behaviour which together constitute a stepwise description of a reference approach to a procedure.

Procedural phase: a group or series of integrally related events or actions that, when combined with other phases, make up or constitute a complete operative procedure.

Step: a component task, the series aggregate of which forms the completion of a specific procedure.

Error: a deviation from optimal performance.

Critical error: a major deviation from optimal performance, which is likely to cause harm to the patient or compromise the safe completion of the procedure [13, 14, 15].

The metrics, therefore, consist of procedural phases involved in a minimally invasive left‐sided colorectal anastomosis. Each phase comprises specific steps required for accomplishment. The importance of the metrics approach in defining these phases and steps is that these are explicit and unambiguous. The procedural step either occurred or did not occur and can be scored as such by an external reviewer with high reliability [16, 17, 18]. Similarly, procedural errors and critical errors were defined associated with particular steps within different phases of the procedure. For errors, behaviours exhibited by the operator may not necessarily in and of themselves lead to a bad outcome or an event with more serious consequences, but their enactment sets the stage or increases the probability for a more serious event to occur or detracts from the efficient and possibly safe execution of the desired procedure. In contrast, a ‘critical error’ is a more serious occurrence and represents operative performance that could either jeopardize the outcome of the procedure or lead to significant iatrogenic damage [11, 13, 14].

**FIGURE 1:**
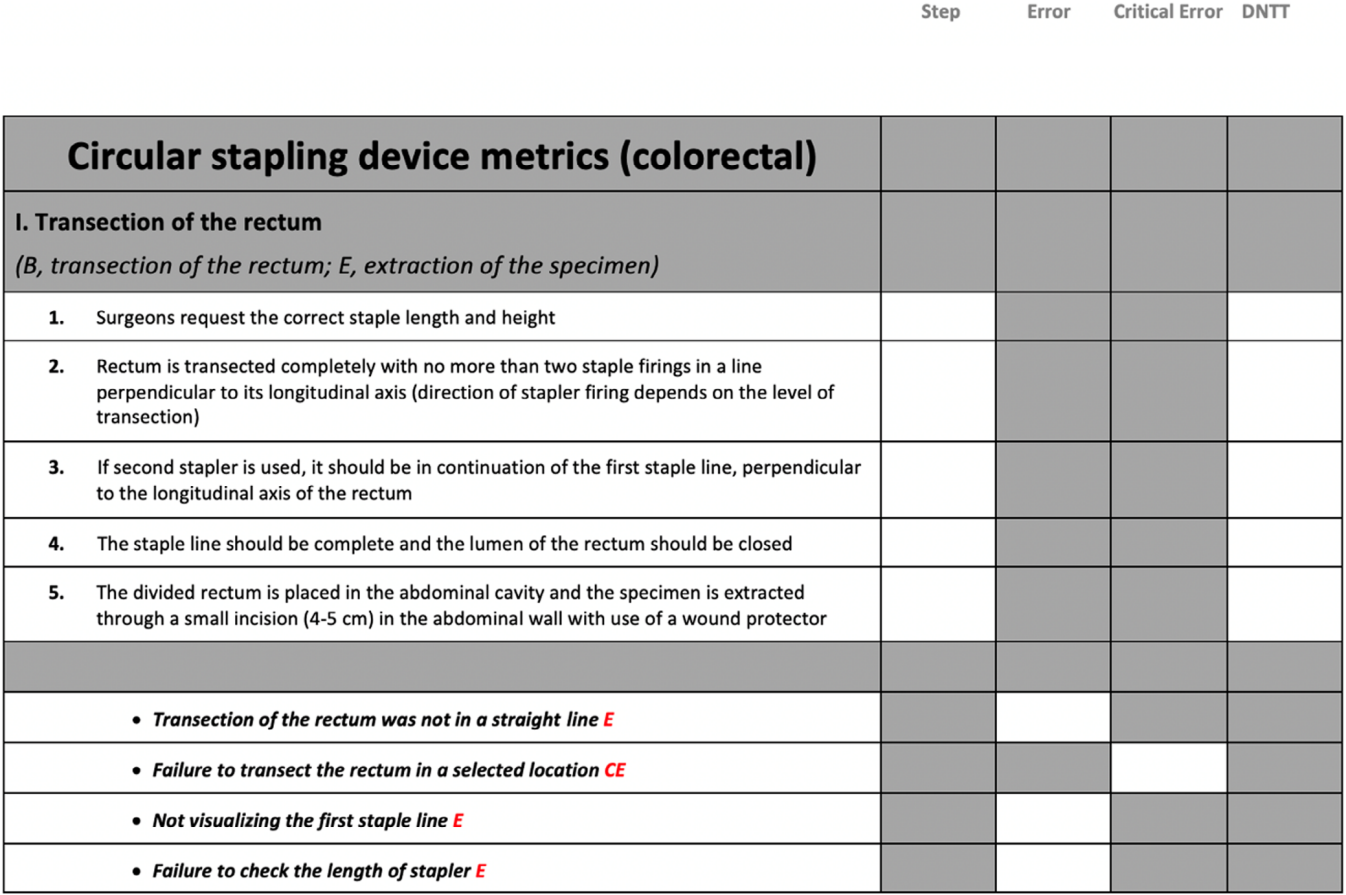
Example of a phase during circular stapling anastomosis in the minimally invasive approach in left‐side colorectal resections that was characterized with steps, errors and critical errors. DNTT, damage to nontarget tissue. Some of the errors/critical errors are shown.

Figure 1 illustrates an example of a procedural phase characterized by circular stapling anastomosis in minimally invasive left‐sided colorectal procedures. In addition to the metrics, valuable knowledge and principles of the operation were compiled, such as the mechanics and science of anastomosis, to facilitate the learning process; these formed the didactic component for the learner during the training process.

Once the Metrics Group had defined the metrics they were then used to score five unedited anonymized circular stapler anastomosis parts of the minimally invasive approach for left‐sided colorectal resection performed by different surgeons with various levels of experience. Scoring was performed by the members of the Metrics Group independently. Any difference in the scoring was discussed in order to identify discrepancies in interpretation or ambiguities in the metric definition. Based on this process, and if agreed upon, changes were made in the metrics, which facilitated the scoring agreement. This process was repeated for each video until the Metrics Group was satisfied with the metrics and they could be scored with a high degree of reliability (i.e. inter‐rater reliability >0.8, which is the internationally agreed gold standard) [19, 20].

### Metrics stress testing (face and content validation) with a modified Delphi approach

Once the metrics for the circular stapling anastomosis for minimally invasive left‐sided colorectal resection had been defined and characterized, face validity and content were verified by a group of experienced colorectal surgeons. An international panel of expert colorectal surgeons was invited to join the Delphi panel [11, 13, 14, 15, 21] to provide a more objective and independent assessment of the metrics. Informed consent was obtained from the Delphi panel members. The panel was chosen for their colorectal surgical experience and their demonstrated educational interests and commitment. The equality, diversity and inclusion principle was adhered to when selecting the Delphi panel members [22].

Sixteen expert colorectal surgeons, including the Metrics Group members from nine countries, a nonvoting behavioural scientist and a nonvoting fellow who is familiar with metrics development in surgical procedures, attended a consensus meeting in Dublin, Ireland on 23 September 2022 (Table 2).

**TABLE 2:** Number of surgeons from each country represented in the Delphi panel.

Country	Number of surgeons
Austria	1
Belgium	3
UK	5
Ireland	1
Switzerland	1
Malaysia	1
Spain	2
Greece	1
Poland	1
Total	16

A brief overview of the project and meeting objectives was presented. Background information regarding PBP training methodology, prior literature demonstrating the validity of this training approach for procedural specialties and the specific objectives of the current Delphi panel were reviewed [23]. Each phase of the procedure, the procedural steps that were included in that phase, and the potential errors were presented. It was also explained that the associated metrics had been developed by the Metrics Group for a reference approach to circular stapling anastomosis for minimally invasive left‐sided colorectal resections. It was acknowledged that the designated reference procedure might not reflect the exact techniques employed by individual Delphi panellists, but that the operative steps presented accurately embodied the essential and key components of the procedure and ‘were not wrong’ [11, 13, 14, 15].

To assess the correlation of the procedural steps, errors and critical errors before and after the Delphi process, changes were analysed with the Pearson chi‐square test (IPM SPSS Statistics for Windows, version 26; IBM Corp., Armonk, NY, USA). A *p*‐value of <0.05 was considered statistically significant.

## RESULTS

The ages of the panel members ranged from 34 to 65 years, and there were five female surgeons. Six panel members were heads of their respective departments and four were full professors affiliated with universities. The combined number of colorectal resections performed or supervised by the Delphi panel was more than 1500 per annum.

The Metrics Group proposed three phases for the circular stapling anastomosis in minimally invasive left‐sided colorectal resection, each with a defined beginning and end (Table 3).

**TABLE 3:** The beginning and end of the different phases of the reference approach to the circular stapling anastomosis for left‐sided colorectal procedures and the changes agreed and voted on by the Delphi panel.

Procedural phase	Title	Phase begins	Phase ends
I	Transection of the rectum	Transection of the rectum	Extraction of the specimen
II	Preparation of proximal colon for anastomosis	Colon is transected completely	Closing the extraction site incision to re‐establish pneumoperitoneum
III	Anastomosis	Move the proximal bowel to pelvis	Check the anastomosis for leakage, e.g. air leak test, rigid or flexible sigmoidoscopy

Some criteria needed to be fulfilled before the circular stapling anastomosis stage. During the Delphi meeting, the Delphi panel suggested and agreed upon two additional conditions (see Method section): the rectal stump should be clean and the surgeon should (have) read the instructions for use for the circular stapling device.

During the Delphi meeting, four steps were added, making a total of 36 steps for the three phases of the circular stapling anastomosis (Table 4). The added steps were ‘Surgeons request the correct staple length and height’ when using a linear stapler in the transection of the rectum (Phase I), ‘Surgeons request for the correct stapler and stapler size’ when using a circular stapler in the preparation of the proximal colon for anastomosis (phase II), ‘Verify verbal communication between the surgical team members before firing the stapler’, ‘Surgeon fire the stapler in a standing position (to stabilize during firing) during anastomosis’ (Phase III). Modifications were made in four steps (Phases I and II) to make the steps more explicit and instructive.

**TABLE 4:** Steps before and after the Delphi meeting.

Procedural phase	Title	Steps before Delphi	Steps after Delphi	Added	Deleted	Modified
I	Transection of the rectum	4	5	1	0	0
II	Preparation of proximal colon for anastomosis	13	14	1	0	1
III	Anastomosis	15	17	2	0	3
Total after three phases		32	36	4	0	4

The Metrics Group identified 40 procedural errors in the three phases, and after the Delphi meeting the total number of procedural errors was 42 (Table 5).

**TABLE 5:** Errors before and after the Delphi meeting.

Procedural phase	Title	Errors before Delphi	Errors after Delphi	Added	Deleted	Modified
I	Transection of the rectum	7	6	0	1	2
II	Preparation of proximal colon for anastomosis	24	24	0	0	1
III	Anastomosis	9	12	3	0	0
Total after three phases		40	42	3	1	0

There were 38 procedural critical errors before and 39 after the Delphi meeting (Table 6).

**TABLE 6:** Critical errors before and after the Delphi meeting.

Procedural phase	Title	Critical errors before Delphi	Critical errors after Delphi	Added	Deleted	Modified
I	Transection of the rectum	7	10	3	0	1
II	Preparation of proximal colon for anastomosis	8	7	0	1	0
III	Anastomosis	23	22	1	2	0
Total after three phases		38	39	4	3	1

Furthermore, the number of procedural steps, errors and critical errors before and after the Delphi changes were highly correlated [Pearson correlation coefficient *r* = 0.974 (95% CI *r* = 0.861–0.994) *p* < 0.001].

On average, there were more procedural steps [before 10.7 (SD = 5.9); after 12 (SD = 6.2)] at the end of the Delphi meeting. The same was observed for errors [before 13.3 (SD = 9.3); after 14 (SD = 9.2)] and critical errors [before 12.7 (SD = 9); after 13 (SD = 8)]. When we compared these differences with Wilcoxon sign rank (two‐tail) tests none of the differences were found to be statistically significant (steps, *Z* = −1.633, *p* = 0.102; errors, *Z* = −0.447, *p* = 0.665; critical errors, *Z* = 0, *p* = 1.0).

After discussion and changes to the metrics incorporated during the meeting, the metrics for circular stapling anastomosis in minimally invasive left‐sided colorectal resection received 100% consensus from the Delphi panel.

## DISCUSSION

Anastomotic complications are common following left‐sided colorectal resection. Among these complications, an anastomotic leak can have devastating consequences for patients' outcomes, including survival rate, cancer recurrence, permanent stoma, negative impact on the bowel and sexual function and long‐term quality of life [1, 24]. Complications also increase the length of hospital stay and place a significant extra resource burden on healthcare institutions [1, 24]. Researchers have been studying the factors associated with anastomotic complications and identifying management strategies to reduce the burden caused by these complications [25]. The circular stapling device is commonly used in left‐sided colorectal anastomosis, in both cancer and benign conditions, but this crucial step of the procedure has not been taught in surgical training. Given that evidence suggests there are gaps in stapling knowledge and a high incidence of technical errors when using a circular stapler, there is an imperative to standardize and define structured training for this critical part of the procedure [2, 3, 4].

More focus is now placed on the surgeon's skill, as evidence now shows that it is strongly linked with patient outcomes [5]. The Metrics Group has identified one scientific approach to structured training in circular stapling anastomosis in minimally invasive left‐sided colorectal resection, namely PBP simulation training. This method makes skill acquisition more objective, transparent and fair. Based on Level 1a evidence, use of the PBP method significantly reduced performance errors by 60% [8].

Using the PBP method, we characterized the performance metrics (procedural phases, steps, errors, critical errors) for circular stapling anastomosis for minimally invasive left‐side colorectal resection. A minimally invasive approach for left‐sided resection is widely practised, but practitioners would find the metrics useful for the open approach. The performance metrics development process was robust and has been used with success in other disciplines [11, 13, 14, 21]. The Metrics Group consisted of three expert colorectal surgeons and individuals who specialize in the PBP methodology, including a senior behavioural scientist with more than two decades of experience in surgical training. Expert engineers working with the circular stapling device were consulted, specifically in relation to instructions for use and technical device handling.

These performance metrics were scrutinized by a panel of expert colorectal surgeons from different European countries and a renowned minimally invasive expert surgeon from an academic centre in Malaysia.

During a minimally invasive approach to left‐sided colorectal resection, surgeons have variations of practice when performing circular stapling anastomosis. The performance metrics presented in the Delphi meeting aimed to outline a standardized approach suitable for learners. Minor modifications were made during the Delphi meeting to make the performance metrics more explicit and instructive. Some general principles, for example stapling technologies, will be provided as didactic to the trainees in addition to the metrics.

The pre‐ and post‐Delphi metrics were highly correlated (Tables 3, 4, 5, 6). After incorporating the changes suggested by the Delphi panel, voting was obtained at the end of the discussion of each phase. All of the procedural phases received unanimous agreement.

Anastomotic complications, particularly leaks, are among the most feared complications in colorectal surgery. The anastomotic part of the procedure is performed towards the end of an operation; potentially, issues of fatigue and concentration may be introduced at this crucial part of the operation. A successful operation also depends on the skills of the operating team, not only the lead surgeon. This is important, as often the introduction of the circular stapling device is performed by more junior surgical team members.

During the Delphi meeting, the panel members recognized the knowledge gap and training needed in the use of the circular stapling device. Some valuable additional comments were made and incorporated into the performance metrics, such as ‘Surgeons request for the correct stapler and stapler size’ and ‘Verify verbal communication between the surgical team members before firing the stapler’.

The PBP approach to characterize these three phases of circular stapling anastomosis during a crucial part of a minimally invasive approach to left‐sided colorectal resection allows surgeons to learn the steps with explicit performance instructions about what to do and, possibly more importantly, what not to do. The PBP method affords performance assessments where the metrics are used to provide feedback to learners that are objective, transparent, explicit, constructive and formative. The errors and critical errors that were described would further enhance training.

The proposed metrics are for a standard and straightforward procedure. The aim is to provide a structured stepwise approach to use of the device during this segment of the procedure. We do, however, appreciate the variety of practices; for example, when making the purse‐string for the proximal end of the colon, a purse‐string applicator can be used instead of a manual purse‐string, as detailed in our metrics.

## CONCLUSION

During a minimally invasive approach to left‐sided colorectal resection, circular stapling anastomosis can be broken down into procedural phases and steps, with errors and critical errors known as performance metrics. Data from a large group of expert colorectal surgeons from Europe provided evidence to support the face and content of these metrics. We consider the metrics essential for developing structured training using circular stapling anastomosis in a minimally invasive approach to left‐sided colorectal resection. Further development of these metrics is vital to guide the training curriculum and assessment.

## AUTHOR CONTRIBUTIONS

**Samson Tou:** Conceptualization; investigation; funding acquisition; writing – original draft; methodology; validation; writing – review and editing; project administration; data curation; supervision; resources; visualization; formal analysis. **Anthony G. Gallagher:** Conceptualization; investigation; funding acquisition; methodology; validation; writing – review and editing; visualization; formal analysis; project administration; data curation; supervision; resources. **Gabriele Bislenghi:** Investigation; writing – review and editing. **Rui Farinha:** Investigation; methodology; writing – review and editing. **Albert Wolthuis:** Conceptualization; investigation; funding acquisition; methodology; validation; visualization; writing – review and editing; project administration; supervision; resources.

## FUNDING INFORMATION

Medtronic (Surgical Division) provided the educational grant for this study but did not influence the selection of the experts, the design and conduct of the research, data collection, analysis or the preparation of the manuscript.

## CONFLICT OF INTEREST STATEMENT

ST received education grants from Intuitive Foundation and Medtronic. AGG holds education research grants from Medtronic (Dublin, Ireland), AO Education Institute (Davos, Switzerland), and the Arthroscopic Association of North America (Chicago, USA) to investigate metric‐based education and training.

## ETHICS STATEMENT

All participants provided informed consent prior to participating in the study, and the study protocol was approved by the institutional review board at the University of Leuven.

## Data Availability

The data that support the findings of this study are available from the corresponding author upon reasonable request.

## Appendix

Circular stapling device in colorectal anastomosis performance metrics CAPITAL A Study Collaborative: Felix Aigner (Hospital of St John of God, Graz, Austria); Gabriele Bislenghi (University Hospital Leuven, Leuven, Belgium); Nicola Eardley (Countess of Chester Hospital NHS Foundation Trust, Chester, UK); Rui Farinha (ORSI Academy, Melle, Belgium); Christina Fleming (University of Limerick Hospital, Ireland); Anthony Gallagher (ORSI Academy, Melle, Belgium); Dieter Hahnloser (Lausanne University Hospital, Lausanne, Switzerland); Seon‐Hahn Kim (University of Malaya Medical Centre, Kuala Lumpur, Malaysia); Brendan Moran (Hampshire Hospitals NHS Foundation Trust, Basingstoke, UK); Guglielmo Niccolò Piozzi (Portsmouth Hospitals University NHS Trust, Portsmouth, UK); Gabrielle van Ramshorst (Ghent University Hospital, Ghent, Belgium); Rosa M. Jimenez Rodriguez (Hospital Universitario Virgen del Rocio, Seville, Spain); Ines Rubio‐Perez (Hospital Universitario La Paz, Madrid, Spain); Samson Tou (University Hospitals of Derby and Burton NHS Foundation Trust, Derby, UK); Ioannis Virlos (Metropolitan General, Athens, Greece); Janindra Warusavitarne (St Mark's Hospital, Harrow, UK); Albert Wolthuis (University Hospital Leuven, Leuven, Belgium); Marek Zawadzki (Faculty of Medicine, Wroclaw University of Science and Technology, Wroclaw, Poland).
